# Acupuncture in the Treatment of a Female Patient Suffering from Chronic Schizophrenia and Sleep Disorders

**DOI:** 10.1155/2016/6745618

**Published:** 2016-12-22

**Authors:** Peggy Bosch, Sabina Lim, Sujung Yeo, Sook-Hyun Lee, Heike Staudte, Maurits van den Noort

**Affiliations:** ^1^Donders Centre for Cognition, Radboud University Nijmegen, Montessorilaan 3, 6525 HR Nijmegen, Netherlands; ^2^Psychiatric Research Institute, LVR-Klinik Bedburg-Hau, Bahnstraße 6, 47551 Bedburg-Hau, Germany; ^3^Research Group of Pain and Neuroscience, Kyung Hee University, No. 47 Gyeonghuidae-Gil, Dongdaemun-Gu, Seoul 130-701, Republic of Korea; ^4^Department of Acupuncture & Meridian of Oriental Medicine, Sang Ji University, 83 Sangjidae-gil, Wonju 26339, Republic of Korea; ^5^Brussels Institute for Applied Linguistics, Free University of Brussels, Pleinlaan 2, 1050 Brussels, Belgium

## Abstract

*Background*. The use of acupuncture in the treatment of sleep disorders in patients with chronic schizophrenia is investigated.* Case Presentation*. We report the case of a 44-year-old female outpatient of German origin who had been suffering from long-term schizophrenia and sleep disorders. The patient was treated with manual acupuncture weekly for 12 weeks, and a psychological assessment was performed before, immediately after, and three months after the acupuncture treatment period. In addition, actiwatch data were collected for 14 days both before and after the acupuncture treatment period.* Conclusion*. Acupuncture treatment led to a decrease in general psychopathology, less severe sleep problems, and markedly improved cognitive functioning (working memory) in the patient; however, the positive and the negative symptoms remained stable. The actiwatch data revealed a beneficial effect of acupuncture, showing better sleep latency, a trend towards better sleep efficiency, and a decrease in the number of minutes that the patient was awake during the night after acupuncture treatment. In sum, this study showed that acupuncture might be beneficial in the treatment of sleep disorders in patients suffering from chronic schizophrenia, but future, large, randomized (placebo), controlled, clinical trials are needed in order to replicate the present preliminary findings.

## 1. Introduction

Schizophrenia is a chronic and severe psychiatric disorder [1] and it occurs in about 0.5% of the world's population [2]. Patients with schizophrenia suffer from positive symptoms, negative symptoms, and cognitive deficits [3], of which working memory problems are considered a central cognitive impairment [4]. In addition to those characteristic symptoms, patients also frequently report sleep disorders [5].

A standard treatment for patients with schizophrenia is pharmacotherapy [6]. However, although such therapy has been reported to have many successes, the number of adverse effects associated with it is large [7], and noncompliance is always an important area of concern [8]. For instance, in previous research, a 47% nonadherence rate to pharmacotherapy was found in patients suffering from schizophrenia [9]. As a result, in addition to pharmacotherapy, nonpharmacological interventions, such as transcranial magnetic stimulation (TMS) [10], transcranial direct current stimulation (tDCS) [11], and convulsive therapy [12], are increasingly being used in the treatment of schizophrenia.

One relatively new nonpharmacological intervention technique in Western psychiatry is acupuncture, which is an old Eastern medicine treatment technique [13]. To date, not much research has been conducted on the use of acupuncture as an add-on intervention technique in the treatment of patients suffering from schizophrenia [13], and almost no research at all exists on the use of acupuncture in the treatment of their comorbid sleep disorders [14]. However, the scarce research to date shows sleep-ameliorating effects of acupuncture in the treatment of sleep disorders in schizophrenia [13]. Therefore, in this study, the use of acupuncture for the add-on treatment of comorbid sleep disorders in a patient with chronic schizophrenia is investigated further by using both psychological-test and actiwatch data. Our hypothesis is that 12 weekly acupuncture treatment sessions will improve the positive and the negative symptoms, alleviate the subjective and the objective sleep problems, and improve the cognitive functioning of a patient suffering from chronic schizophrenia.

## 2. Case Presentation

A 44-year-old female outpatient of German origin, who had been suffering from long-term schizophrenia (length of illness: 16 years) and (subjective) sleep disorders, was the subject of this study. The patient was diagnosed by experienced psychiatrists and was found to be suffering from paranoid schizophrenia, F20.0 according to the International Classification of Diseases-10 [15], and schizophrenia according to the Diagnostic and Statistical Manual of Mental Disorders-V [16]. The patient had normal intelligence (IQ = 88, as measured with the MWTB test) [17], and she had finished “Hauptschule” education, which is secondary school in Germany. The patient was on medication during the whole study. She used amisulpride (200 mg in the morning), olanzapine (5 mg at night), and pramipexole (0.70 mg in the evening).

The patient was suffering from severe recurrent psychosis involving repetitive delusions. During psychotic episodes, she would have the feeling that another person, that is, the devil, was residing in her. Moreover, she was suffering from severe sleep disorders, including insomnia, frequent awakenings, and nightmares. She was also suffering from social dysfunctioning and restlessness and had had the feeling of being different within, as well as outside, the body; sometimes, she exhibited catatonic behavior. During psychosis, she would have the disturbing impression that things, such as doors and her own hands, had changed and become larger. Moreover, she claimed to feel the soul of her best friend. She was also suffering from severe anxiety with mood changes from disturbed laughing to extensive crying. Sometimes, she would feel as if someone had pushed away her feet when she was seated, and, sometimes, she would see different people present in the bodies of her friends. Finally, she would have the feeling of being observed all the time.

**Figure 1:**
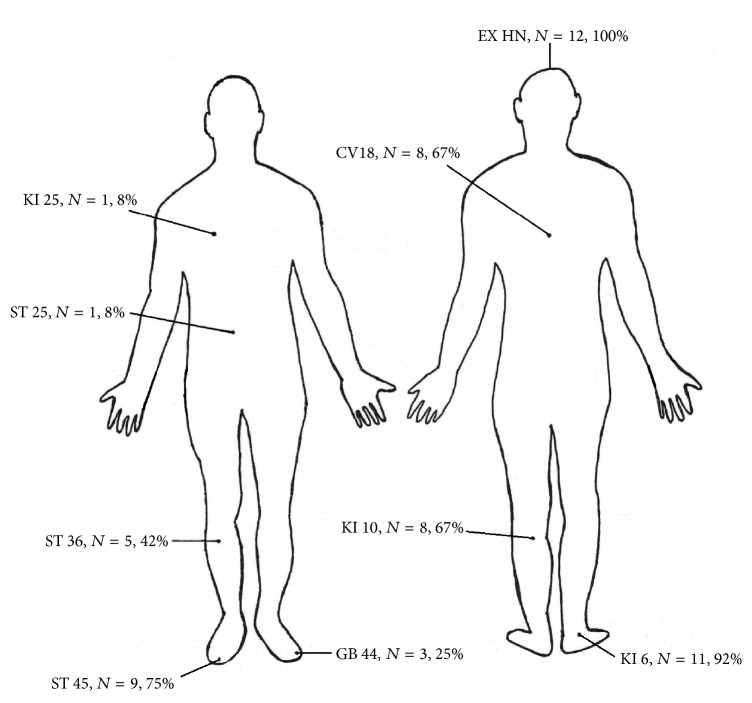
An overview of the acupuncture points (including the absolute frequency numbers and percentage scores) that were used during the 12 weekly acupuncture treatments of our 44-year-old female outpatient suffering from chronic schizophrenia and sleep disorders.

Approval was received from the local ethics committee (Ärztekammer Nordrhein, number 2008331) for this clinical case study; moreover, the study is part of a larger project that has officially been registered under number NTR3132 at the Dutch Trial Register. The following study protocol was used: (1) a psychological assessment was conducted using the Pittsburgh Sleep Quality Index (PSQI) [18] in order to measure subjective quality of sleep, the digit span forward and backward [19] in order to measure simple working memory performance [20], the letter-number sequencing task [19] in order to measure complex working memory performance [20], and the Positive and Negative Syndrome Scale (PANSS) [21] filled in by her psychiatrist in order to monitor the positive and the negative symptoms. Her psychiatrist had had more than 20 years of psychiatric experience in the LVR-Klinik Bedburg-Hau and had been trained in using the PANSS. Moreover, the psychiatrist was not involved in and/or informed about our research project. In addition, the patient wore an actiwatch (Type: Actiwatch Spectrum Plus, http://www.actigraphy.com/devices/actiwatch/actiwatch-plus.html) for 14 days, 24 hours a day, in advance of the clinical nonpharmacological intervention in order to collect data on the following seven sleep parameters: “sleep efficiency,” “sleep latency,” “absolute actual sleep time,” “absolute actual wake time,” “relative actual sleep time,” “relative actual wake time,” and “assumed sleep” (meaning the difference between the end of sleep and the start of sleep). (2) Then, after careful diagnosis [22] by a licensed Oriental medical practitioner with more than five years of clinical experience [23], the patient received individualized manual acupuncture in accordance with traditional Chinese medicine principles [22]. Single-use stainless-steel needles (Type: AcuPro C, Wujiang City Cloud & Dragon Medical Device Co., Ltd., China) were used. The patient was treated weekly in the clinic for twelve consecutive acupuncture treatments, and each acupuncture treatment lasted about 60 minutes (for a detailed overview of the exact acupuncture points and frequency that were used during the 12 weekly acupuncture treatments, we refer the reader to Figure 1).

For each separate acupuncture needle used in the treatment, the needle was inserted and* deqi* [24] was achieved. The needle was then left in place for one hour [25], after which it was taken out. (3) After the 12 weeks of treatment, a second psychological assessment was completed using the same psychological tests that had been used in the first psychological assessment. Moreover, again, she put on and wore an actiwatch for 14 days, 24 hours a day. (4) Finally, three months after having finished the acupuncture treatment, a third psychological assessment was completed using the same psychological tests as in the first and the second psychological assessments in order to investigate possible long-lasting treatment effects.

As can be seen in Table 1, the psychological assessment results show no differences in the PANSS positive and the PANSS negative subscale scores before acupuncture, immediately after the acupuncture treatment period, and three months after the acupuncture treatment period, with all scores remaining stable at 7. The PANSS psychopathology score was observed to have decreased from 38 before acupuncture to 30 immediately after the acupuncture treatment period but to have increased to 33 at follow-up, three months after having finished acupuncture treatment. The PANSS total score was observed to have decreased from 52 before acupuncture to 44 immediately after the acupuncture treatment period but to have increased to 47 at follow-up. The PSQI total results showed a decrease from 9 before acupuncture to 3 immediately after the acupuncture treatment period, but, after three months, an increase to 10 was noted. In the literature, three empirically derived factors have been proposed [26], for example, sleep efficiency, perceived sleep quality, and daily disturbances, that according to the authors should be favored over the single factor PSQI total score. However, further research is ongoing [27] and we therefore decided to report all seven components instead of the three, in order to give all necessary information. As can be seen in Table 1, the factor PSQI subjective sleep quality decreased from 1 before acupuncture to 0 after acupuncture, and it then increased to 3. The factor PSQI sleep latency first decreases from 3 to 1 and then increases back to 2 at follow-up. The factor PSQI sleep time increases from 0 to 1 in score (indicating a more problematic sleep time) after acupuncture but then decreases again. The factor PSQI sleep efficiency does not change between T1 and T2 but increases to 3 at follow-up. The factor PSQI sleep disorders decreases after acupuncture and then stays that way. The factor PSQI sleep medication decreases after the first measurement and does not change again after that. The factor PSQI daytime sleepiness remains at zero until it increases to 1 at follow-up. The factor PSQI subjective time to fall asleep in minutes decreases markedly after T1 in which the patient estimated that it took approximately 60 minutes to fall asleep each day. After acupuncture, she fell asleep after only thirty minutes and this further improved to 4.5 minutes at follow-up. The factor PSQI subjective sleep duration in hours consisted of the total sleep time that the patient filled in on the form, and it decreased dramatically after acupuncture and stayed approximately the same at follow-up. The digit span results showed an increase from 7 before acupuncture to 10 immediately after the acupuncture treatment period and remained stable at 10 three months after having finished the acupuncture treatment. Finally, the letter-number sequencing results showed an increase from 2 before acupuncture to 6 immediately after acupuncture treatment and remained relatively stable and even slightly increased up to 7, three months after having finished the acupuncture treatment.

Moreover, as can be seen in Table 2, the actiwatch results before acupuncture treatment versus those after acupuncture treatment showed a statistically significant decrease in the scores for “sleep latency” (*t* = −3.25, *p* = 0.006). Moreover, a certain trend towards significance was found for the sleep parameters “sleep efficiency” (*t* = 1.89, *p* = 0.08) and “absolute actual wake time” (*t* = −1.89,* p* = 0.08). More specifically, “sleep efficiency” increased from 80.31% to 85.64% while “absolute actual wake time” went down from 107.30 minutes to 73.38 minutes after acupuncture treatment. Finally, no statistically significant change or trend towards significance was found for “absolute actual sleep time” (*t* = −1.09, *p* = 0.30), “relative actual sleep” (*t* = 1.58, *p* = 0.14), “relative actual wake” (*t* = −1.58, *p* = 0.14), and “assumed sleep” (*t* = −1.70, *p* = 0.11) (all raw actiwatch data for our patient are presented in Supplementary Table 3 in Supplementary Material available online at http://dx.doi.org/10.1155/2016/6745618).

## 3. Discussion

In this study, the use of acupuncture in the treatment of comorbid sleep disorders in a patient with chronic schizophrenia was investigated by using both psychological-test and actiwatch data. Our results show that, in line with our hypothesis, acupuncture treatment had an immediate effect on the patient's general psychopathology, which showed a decrease after acupuncture treatment. Also, three months after the acupuncture treatment period, the general psychopathology remained lower than it was before acupuncture treatment, although it had increased slightly again. Moreover, the positive and the negative symptoms remained stable; at all three assessments, before, immediately after, and three months after acupuncture treatment, an extremely low PANSS score of 7 was observed, indicating that her psychiatrist had assessed the patient as having no positive and negative symptoms at all. Because of this extremely low score at the beginning of the study, we were unable to measure any beneficial effect of the acupuncture treatment. Note that the psychiatrist had had more than 20 years of psychiatric experience and had been trained in using the PANSS; therefore, mistakes in using the scale seem unlikely. A more likely explanation for this extremely low PANSS score of 7 for the positive and the negative symptoms seems to be that our patient received acupuncture treatment in addition to her standard (pharmacological) treatment, which was already successfully treating her positive and negative symptoms. In future research, patients with higher starting scores for the positive and the negative symptoms should, therefore, be included in order to investigate the effects of acupuncture treatment on the positive and the negative symptoms and in order to be able to test the hypothesis that 12 weekly acupuncture treatment sessions improve the positive and the negative symptoms, which could not be verified in the present case study.

However, as we expected, the subjective experience with sleep problems showed an immediate effect after acupuncture treatment. After the acupuncture treatment, the total PSQI [18] score had decreased to below the clinically used original cut-off score of 5 [18], as well as the more recently used higher cut-off score of 6 [28], meaning that the patient was experiencing no sleep disorders. However, three months after having finished the acupuncture treatment, this effect was lost, and the score was observed to have increased to the level before acupuncture treatment, showing that this improvement in subjective sleep experience is not a sustained effect.

Logically, the following subscores were found, which illustrate the above findings. The subjective sleep quality improved after acupuncture but decreased again at follow-up. The same pattern was visible for her sleep latency. Interestingly, she subjectively fell asleep increasingly fast during the whole process, but at follow-up she slept less yet stayed in bed for 11 hours, explaining the score of 2 for sleep latency. Our patient reduced her sleep time after acupuncture and maintained a healthy score at follow-up. Although the PSQI score for sleep duration did not score negatively at T1, it seems healthier to sleep approximately 7 hours instead of 10 to 12 [29]. Sleep efficiency was good at T1 and T2; only at follow-up it was bad. Without informing the study coordinator, the patient stopped using her medication for sleep (without prescription) during the acupuncture period and did not start again after that. Daytime sleepiness was OK but increased slightly at follow-up.

Finally, with respect to the cognitive functioning of our patient, as we had hypothesized, the so-called simple [20] and complex [20] working memory performances showed an immediate and sustained effect after acupuncture treatment, with an even stronger effect in complex working memory performance. Again, the explanation for this latter effect seems to be that the beginning level of the patient was very low, with a norm score of 2, for her complex working memory performance, and as a result, the patient could benefit more from acupuncture treatment with respect to her complex working memory performance. On the other hand, her first score of 7 on the simple working memory test was closer to the normal range and more in line with the expectation built upon her education and intelligence. In sum, the working memory performance of our patient revealed that she seemed to benefit markedly from acupuncture treatment and that this positive effect in cognitive functioning seemed to be a sustained effect.

In addition, in line with our hypothesis, the actiwatch results showed an effect of 12 weeks of acupuncture treatment on “sleep latency,” meaning a decrease in latency before sleep onset following bed time. In addition, a trend was visible for “sleep efficiency” and “absolute actual wake time.” Our patient tended to sleep more efficiently after acupuncture treatment and to be awake less (almost 34 minutes on average!) during the night after acupuncture treatment. No effects of acupuncture were found for the “absolute actual sleep time,” “relative actual sleep”/“relative actual wake,” and “assumed sleep.” Overall, an important observation is that the standard deviations of the scores for the sleep parameters were large, showing that the scores for our patient varied greatly over the 14 days. Clinically, this variability is an important finding, showing the importance of measuring the sleep parameters for patients with chronic schizophrenia over a longer time in order to obtain more reliable measurements. This variability might also explain the PSQI finding of total sleep time which was virtually the same at T1 as the actiwatch assumed sleep time but not at T2 where the patient thought she slept 6.5 hours, whereas the actiwatch assumed over 10 hours. It might be difficult for patients to actual guess how long they sleep if the variability is very large.

Taken together, this study showed that actiwatch data can be clinically helpful in determining the exact sleep problems of a patient because actiwatches give “objective” information on specific sleep parameters [30], 24 hours a day, during the entire recording time (in our case, 14 days before and 14 days after acupuncture treatment). As a result, the specific sleep disorders would be clearer than they would have been if only the means of the specific sleep parameters had been calculated. This is because the means can be normal, but, at the same time, the standard deviations can be huge; for instance, regarding the total sleep time, the patient may sleep extensively for several days and not very long on other days, but, by doing so, the mean may still be completely normal. Sleep inventories, such as the PSQI [18], are clinically informative, but we must stress that they give important information on the patient's “subjective” experience with sleep problems, which is as such clinically valuable but that they are not “objective” measures of sleep problems [31]. We should also note that reports in the literature indicate that patients suffering from chronic schizophrenia tend to underestimate their psychopathology [32]. Therefore, the same might be the case with sleep; patients might experience their sleep as normal, although, compared to the normal population, it is not. In sum, the use of both “objective” and “subjective” measures of sleep [31] in patients suffering from chronic schizophrenia seems to have clinical value.

A limitation of the present study is that we had no follow-up actiwatch data, and, as a result, we were unable to determine whether the observed effects on the sleep parameters immediately after acupuncture treatment were also sustained effects. In future research, therefore, the inclusion of follow-up actiwatch data would be useful. Moreover, future studies with large, randomized (placebo), controlled, clinical trials are needed [13] in order to replicate the preliminary acupuncture effects that were found in the present study and in order to answer the question whether acupuncture has an independent effect on improving cognition or whether the improved cognition is secondary to improved sleep. Furthermore, taking a closer look at the gender issue would be particularly interesting [33] in order to investigate whether male patients with chronic schizophrenia and sleep disorders show the same beneficial acupuncture effects as the present female patient suffering from chronic schizophrenia and sleep disorders did. Finally, in future research, the use of electroencephalography (EEG) recordings [34], in addition to actiwatch recordings, would be helpful in order to investigate the neural effects of acupuncture on disturbed sleep in patients with chronic schizophrenia.

## 4. Conclusion

This study showed that acupuncture treatment might be beneficial in the treatment of sleep disorders in patients suffering from schizophrenia. After acupuncture treatment, our patient's general psychopathology had decreased, she was experiencing less severe sleep problems, and her cognitive functioning (working memory) had improved markedly. Furthermore, this study showed that actiwatches, which provide an objective measure of sleep problems, are clinically informative because they can identify sleep problems that would not have been detected by psychological tests (sleep inventories) alone. In this study, we found a beneficial effect of acupuncture: sleep latency was improved after acupuncture treatment, and trends towards better sleep efficiency and a decrease in the number of minutes that the patient was awake during the night were noted. However, replication studies with large, randomized (placebo), controlled, clinical trials are needed in order to verify the preliminary acupuncture effects that were found in the present case study.

## Supplementary Material

Raw actiwatch data for our 44-year-old female outpatient suffering from chronic schizophrenia and sleep disorders before and after acupuncture treatment can be found in Supplementary Table 3. The raw actiwatch data are specified for the following seven sleep parameters: “sleep efficiency,” “sleep latency,” “absolute actual sleep time,” “absolute actual wake time,” “relative actual sleep,” “relative actual wake,” and “assumed sleep” and they contain 14 days of actiwatch recordings before and after acupuncture treatment.

## Figures and Tables

**Table 1:** Psychological assessmentresults for our patient with chronic schizophrenia on the PANSS, PSQI, BDI, digit span, and letter-number sequencing tests before and after acupuncture treatment and at follow-up.

Test	Before acupuncture	After acupuncture	At follow-up
PANSS positive	7	7	7
PANSS negative	7	7	7
PANSS psychopathology	38	30	33
PANSS total	52	44	47
PSQI total	9	3	10
PSQI subjective sleep quality	1	0	3
PSQI sleep latency	3	1	2
PSQI sleep time	0	1	0
PSQI sleep efficiency	0	0	3
PSQI sleep disorders	2	1	1
PSQI sleep medication	3	0	0
PSQI daytime sleepiness	0	0	1
PSQI subjective time to fall asleep in minutes	60	30	4.5
PSQI subjective sleep duration in hours	10–12	6.5	7
Digit span	7	10	10
Letter-number sequencing	2	6	7

**Table 2:** Actiwatch results for our patient with chronic schizophrenia before and after acupuncture treatment.

Actiwatch sleep parameters	Before acupuncture	After acupuncture
Sleep efficiency^1^	80.31 (10.98)^2^	85.64 (2.60)
Sleep latency^3^	24.43 (12.34)	14.04 (8.01)^*∗∗*^
Absolute actual sleep time^3^	583.13 (119.41)	537.34 (108.41)
Absolute actual wake time^3^	107.30 (50.34)	73.38 (30.22)
Relative actual sleep^1^	83.68 (9.92)	88.31 (2.84)
Relative actual wake^1^	16.32 (9.92)	11.69 (2.84)
Assumed sleep^3^	690.43 (92.16) (≈11 hours, 30 minutes)	611.13 (133.46) (≈10 hours, 12 minutes)

^1^In percentages, ^2^standard deviation, and ^3^in minutes; ^*∗∗*^means a statistically significant difference (*p* < 0.01) in score on the paired sample *t*-test between before and after acupuncture.
