# Supplementary material for: Acupuncture in the Treatment of a Female Patient Suffering from Chronic Schizophrenia and Sleep Disorders
Source: Case Rep Psychiatry. 2016 Dec 22;2016:6745618. doi: 10.1155/2016/6745618 (PMC5213811; doi:10.1155/2016/6745618)
Supplement: Supplementary file 1 — Raw actiwatch data for our 44-year-old female outpatient suffering from chronic schizophrenia and sleep disorders before and after acupuncture treatment can be found in Supplementary Table 3. The raw actiwatch data are specified for the following seven sleep parameters: “sleep efficiency,” “sleep latency,” “absolute actual sleep time,” “absolute actual wake time,” “relative actual sleep,” “relative actual wake,” and “assumed sleep” and they contain 14 days of actiwatch recordings before and after acupuncture treatment. [file 6745618.f1.pdf]

Supplementary Material

Supplementary Table 3 Raw actiwatch data for our 44-year-old female outpatient suffering from chronic schizophrenia and sleep disorders before and after acupuncture treatment.

**Actiwatch Sleep Parameters**	**Before Acupuncture**													
Days	***1***	***2***	***3***	***4***	***5***	***6***	***7***	***8***	***9***	***10***	***11***	***12***	***13***	***14***
Sleep efficiency[1]	83.5	82.7	83.9	86.2	84.9	86.8	82.4	45.3	86.3	86.9	66.5	85.7	77.9	85.4
Sleep latency[2]	47	33	13	30	33	7	25	43	4	16	37	23	17	18
Absolute actual sleep time[2]	685	677	590	550	690	614	588	288	548	651	341	684	600	659
Absolute actual wake time[2]	84	109	98	58	89	84	98	274	76	81	121	90	148	95
Relative actual sleep[1]	89.1	86.1	85.8	90.5	88.6	88	85.7	51.2	87.8	88.9	73.8	88.4	80.2	87.4
Relative actual wake[1]	10.9	13.9	14.2	9.5	11.4	12	14.3	48.8	12.2	11.1	26.2	11.6	19.8	12.6
Assumed sleep[2]	769	786	688	608	779	698	686	562	624	732	462	774	748	754
**Actiwatch Sleep Parameters**	**After Acupuncture**													
Days	***1***	***2***	***3***	***4***	***5***	***6***	***7***	***8***	***9***	***10***	***11***	***12***	***13***	***14***
Sleep efficiency[1]	84.6	83.4	83.6	86.1	84.9	88.8	84.4	83.5	88.1	89	80.7	85.8	90.7	85.4
Sleep latency[2]	15	9	10	24	17	10	9	32	19	20	19	6	1	6
Absolute actual sleep time[2]	440	649	588	454	508	541	667	364	682	548	613	386	408	678
Absolute actual wake time[2]	58	113	91	47	70	56	114	34	68	45	128	58	39	110
Relative actual sleep[1]	88.4	85.2	86.6	90.6	87.9	90.6	85.4	91.5	90.9	92.4	82.7	86.9	91.3	86
Relative actual wake[1]	11.6	14.8	13.4	9.4	12.1	9.4	14.6	8.5	9.1	7.6	17.3	13.1	8.7	14
Assumed sleep[2]	498	762	679	501	578	597	781	398	750	593	741	444	447	788

[1] in percentages, [2] in minutes
